# The intersection of race and femininity in the classroom

**DOI:** 10.3389/fpsyg.2023.1139320

**Published:** 2023-09-05

**Authors:** Naomi S. Faber, Monnica T. Williams

**Affiliations:** ^1^Department of Psychology, Bryn Mawr College, Bryn Mawr, PA, United States; ^2^School of Psychology, University of Ottawa, Ottawa, ON, Canada

**Keywords:** racism, intersectionality, diversity, feminism, gender, allyship, toxic femininity

## Abstract

This vignette told in eight graphic panels illustrates a story about how emotional responses associated with White femininity are used to derail a classroom discussion about racial injustice in a university setting. The panels show how this weaponization of femininity occurs and how it shields those who wield it from external criticism while centering themselves in conversations about race. Women of other races typically cannot access this psychological tactic, thus it constitutes a strategic intersectional use of race, psychology, and privilege to access a power position. In offering suggestions on how to respectfully engage in situations in which racial injustice is a topic of discussion, we unveil how failure of emotional regulation is part of the core psychological framework that leads to these kinds of power dynamics.

## Highlights

- Race as a discussion topic is actively avoided or derailed by White students; women strategically use their gender to this end.
- There is societal deference toward the visible distress made by a White woman in public that is not afforded other racial groups.
- This knowledge is used strategically by White women with and without their conscious intent to decenter people of color and center themselves.

“Between the intersection of whiteness, womanhood and patriarchy stands the white woman. She is seen as vulnerable, feminine, and emotional. A figure in need of protection” – Phipps (2021).

## 1. Introduction

The intersection of race and femininity is a complex and multifaceted topic that has received increasing attention in recent years. Research has shown that the actions of women of color are perceived differently than those of White women in the same contexts (McCormick-Huhn and Shields, 2021). Women of color are more likely to experience sexual harassment, for example, and may be more vulnerable to other forms of violence and abuse due to the juxtaposition of their race and gender identities (Cassino and Besen-Cassino, 2019). Studies have also shown that women of color often face unique challenges and discrimination due to this intersection of race and gender. For example, a nationally representative survey (*N* = 2,009) found that Black and Latina women are likely to receive less support after reporting harassment than their White counterparts (Raj et al., 2021). Further, White women are less likely to give aid to women of color, as illustrated in a study by Katz et al. (2017) on bystander responses to a Black woman at risk for sexual assault when incapacitated (*N* = 160). White women in contrast, may be more likely to be able to rely on their gender to protect them from certain types of violence and harassment, while women of color may be more vulnerable to these types of harms due to their race (Accapadi, 2007; Butler et al., 2022).

These are however not the only ways that race, and gender intersect. The knowledge that an individual develops growing up socialized as White results in an understanding that Whiteness grants power that others of different races cannot easily access. Growing up female and White, an individual will have observed a deference granted to specific emotional states that are unique to her race and gender. This societal deference provides White women with a set of tools that can be used to garner sympathy in high-risk situations, shielding her physically and mentally from external threats (Armstrong, 2021; Phipps, 2021; Butler et al., 2022). These tools of sympathy and protection of the psyche are then wielded consciously or unconsciously in moments where the user finds herself under physical threat or emotional stress (Butler et al., 2022). Recent examples of how White women have attempted successfully and unsuccessfully to use their Whiteness and their femaleness to protect themselves from perceived threats by people of color have become widely available in the media (Armstrong, 2021). These scenarios have emerged in a spectrum of examples from police encounters in which a White woman attempts to blame-shift her shoplifting crime onto a Black woman, to an incident in which a White woman attempts to have a Black man arrested after he asks her to leash her dog while bird watching (Armstrong, 2021). Indeed, many people of color report experiencing more racial aggression from White women than White men (e.g., Blow, 2020; Williams, 2021).

When students are coming into contact with individuals from diverse cultures and are exposed to course material about privileged social identities, blindness to racial privilege can become a source of classroom conflict that exposes fault lines that have been previously hidden (Boatright-Horowitz and Soeung, 2009; Bergkamp et al., 2022; Todd et al., 2023).

### 1.1. Positionality

The authors of this paper have witnessed this phenomenon occurring at universities in different countries and from both sides of the classroom. The first author is a Black German American woman attending a competitive liberal arts college in the United States. The second author is a tenured psychology professor and research chair of mental health disparities who studies racism and psychopathology at a bilingual French-English urban university in Canada. This paper is informed by the authors’ lived-experience, expertise in the subject matter, scientific research, and the scholarly literature.

### 1.2. Classroom disruption

As an example, in the summer of 2022, one of the authors was curating a class for approximately 25 graduate students at a Canadian university. There was a section on cultural competence in psychology, and the discussion centered on the experiences of people of color and societal privilege. Instead of accepting the sources and experience of the instructors, four White students made the point that White French-speaking Canadians are also the subject of discrimination by English-speaking Canadians, and that they would like to find out how this topic would be integrated into the material. They furthermore expressed a general disbelief that there were differences between the opportunities of people of color and White people in Canada, even after being confronted with data and evidence showing the contrary (e.g., Cénat et al., 2021). The topic of discussion became negatively focused on the students of color in the class, their opportunities, and qualifications, and by implication, their humanity. The students of color felt unsafe and insulted, and expressed this to the instructor after the class was over.

When these White students were asked by the instructor to save further discussion for a later date they refused, continuing to monopolize class time, and then after the class they posted their opinions on the class discussion forum and complained to the administration that their academic freedom was being suppressed. They had managed to halt the lesson and prevent the course material from being covered in the allotted time. In particular, some White female students spearheaded the backlash, often becoming emotional or even insulting during discussions. One of the White female students asked a guest lecturer how she deals with “being advantaged on the basis of being a Black person,” although this was not expressed anywhere in the lecture; rather the material covered societal advantages of Whiteness. It eventually became necessary to bring in two additional instructors and increase the class time to resolve the conflict. Meanwhile, the students of color were asking why the course was being derailed to coddle the White students.

This kind of classroom disruption during discussions on topics of race are an oft observed issue that occurs around the intersection of gender and race (Sue et al., 2009a; Sue, 2013). Sue et al. (2009b) conducted a qualitative study of racially-charged classroom conversations with eight White professors. Loss of control over the classroom was a major theme, as was uncertainty for how to address these conflicts, as “White students crying in response to a difficult dialogue was a common observation” (p. 1099). Likewise, in their focus group study (*N* = 14), Sue et al. (2009a) note that “the most common” reaction to difficult classroom discussions about race was crying” (p. 187).

## 2. Powerplay in the classroom: graphic representation

**Figure 1:**
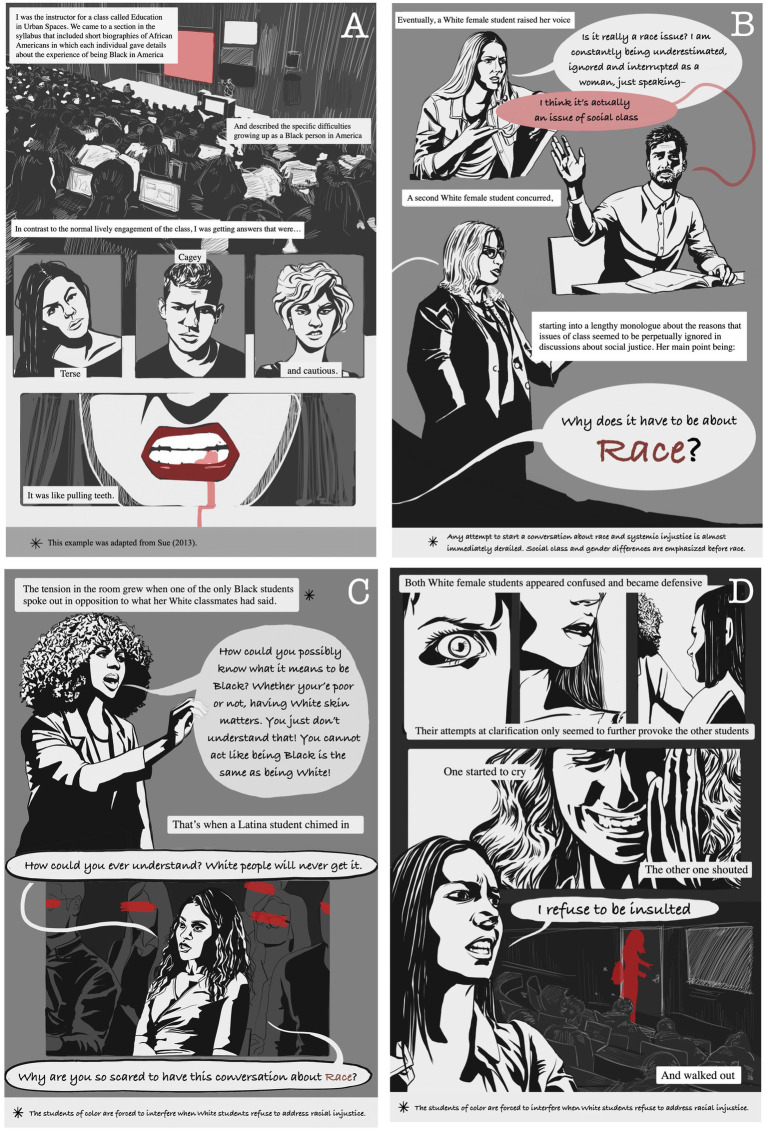
Powerplay in the classroom: weaponizing femininity in a discussion about race. **(A–D)** Instructor notices differing reactions between White and non-White students to a discussion about race.

**Figure 2:**
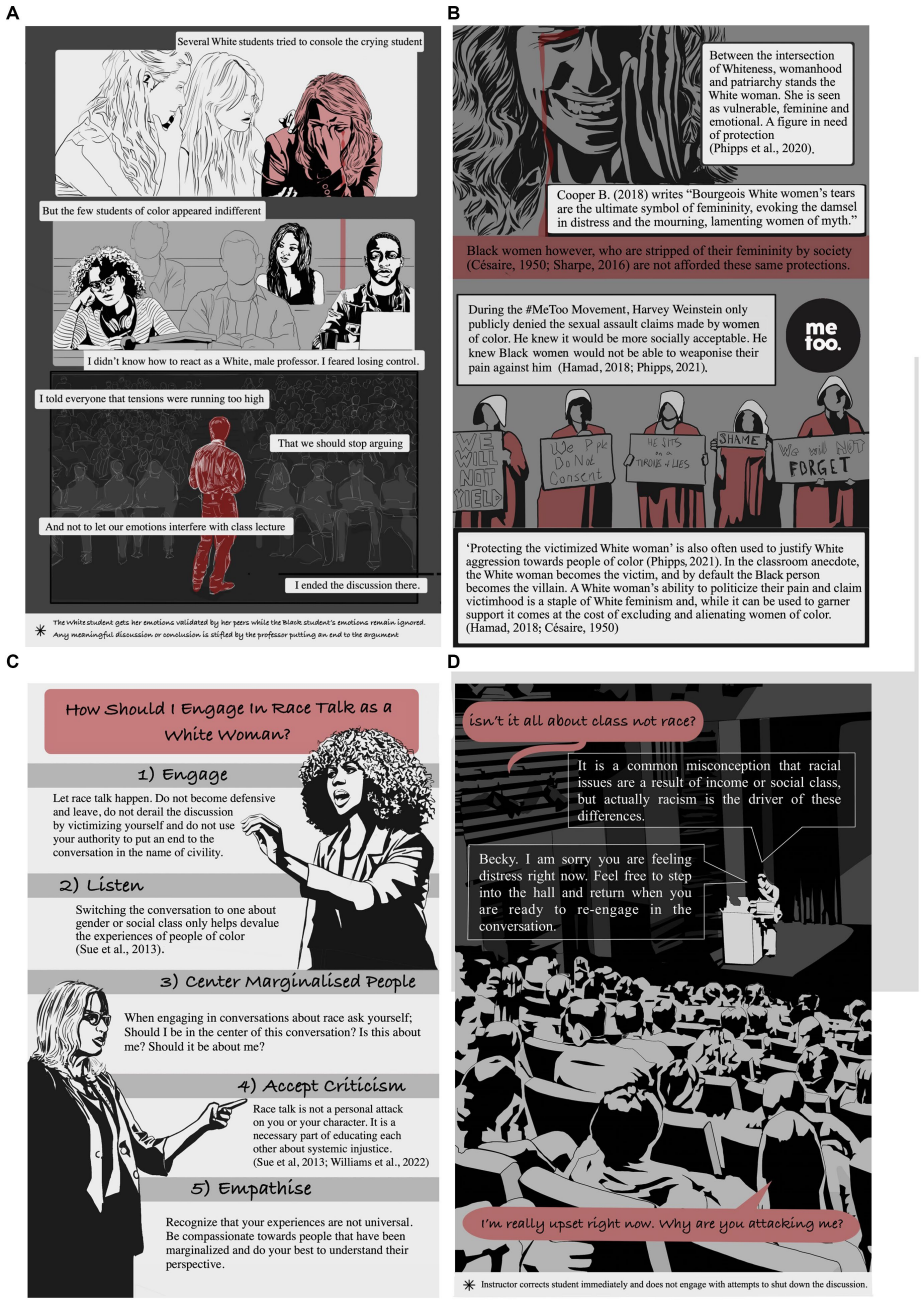
Powerplay in the Classroom: Loss of control in a discussion about race - analysis and recommendations **(A)** Instructor closes discussion which has been derailed. **(B)** Politicization of trauma is possible in White concepts of femininity, but more difficult for women of color. **(C)** Suggestions for engaging in discussion about race. **(D)** Lecturer corrects students and maintains control of the lesson.

The college setting is a particularly volatile venue in which intersectionality between race and gender often comes to a head (Sue et al., 2009a; Sue, 2013). In the vignette illustrated in Figures 1, 2, based on classroom situation described by Sue (2013), a White university professor attempts to address the topic of racial injustice in a classroom when his lecture is derailed by several White students. Students of color are forced to interfere when the White students refuse to address racial injustice being described by the professor. The professor is surprised by the antagonism and his inaction allows the argument to escalate.

Several White students employ strategies to put an immediate end to the conversation and leave the arguments of the Black students unaddressed (Figures 1A–C). Some White women students in the class take personal offense then one begins to cry (Figure 1D) thereby refocusing the center of attention and creating a visible classroom disruption. Another White female student becomes defensive and leaves, putting an immediate end to the discussion. The emotions of the White female students are validated by her peers while the Black student’s emotions remain ignored (Figure 2A) (e.g., Cooper, 2018). The Black student furthermore is villainized through the actions of the White student and her argument is viewed as invalid.

This is a scene that is not new. Historically, Black women have been denied their femininity by society and are not afforded the same protections as White women (Césaire, 1950; Sharpe, 2016; Truth, 2020; Williams, 2021; Butler et al., 2022). This history influences how Black students are seen by their peers in many settings including the college classroom. The discussion topic in this case has created a racial divide in the class; in such cases White students and students of color have different levels of power that can be applied to promote understanding, and students of color would have needed an ally. An ally is a person who advocates for the inclusion of marginalized people groups, not as a member of that group but in solidarity with its struggle and perspective (Williams et al., 2021). In this case, any meaningful discussion or conclusion is stifled by the professor who has become uncomfortable by the antics of the problematic White students, putting an end to the argument after exhibiting an unwillingness to demonstrate allyship or courage on behalf of the students of color in his classroom.

## 3. Discussion

White women, as in the graphically depicted situation in Figures 1, 2, have the ability to weaponize their pain and claim victimhood in situations where they feel threatened, an option that has not been accessible for women of color (Truth, 2020; Phipps, 2021). Certainly, other types of identities will also moderate this ability (e.g., age, income, religion, disability), but all things being equal, race and gender typically advantage White women in these situations. This use of vulnerability has been described as a staple of White feminism leading to discussions about ‘toxic femininity’ (Accapadi, 2007; Hamad, 2019; Williams, 2021), whereby patriarchal social norms are enacted by White women to gain power in certain situations (patriarchal femininity, Patton, 2018; McCann, 2022). Underscoring feminine stereotypes about weakness and vulnerability can help enlist assistance by more powerful actors. Although it can be used to enlist support for important issues (i.e., as in the #MeToo Movement) the cost of this kind of enactment is the exclusion and alienation of women of color, whose cultural concept of femininity may be less inclusive of weakness or vulnerability (Césaire, 1950; Hamad, 2019). The idea of the ‘White woman’, who is seen as a victim in need of protection, has been used in the past to justify aggression, anger, and violence toward people of color (Patton, 2018; Phipps, 2021; Butler et al., 2022). Historically, a colonial-era pretext for subjugation and violence by White men against people of color has been protection of the damsel-in-distress.

One of the most galvanizing historic examples of such aggression is the 1955 murder of Emmet Till, a 14-year-old African American boy who was horrifically murdered by White men after being falsely accused of whistling at a White woman (Pérez-Peña, 2017). This type of violence was not uncommon in the segregated South in that era; however, in this instance, the widely publicized photos of his mutilated body resulted in such national notoriety, that to this day the commemoration markers of his death are continuously vandalized (Threatte, 2016; Pérez-Peña, 2017; Paz, 2021). Likewise, the Tulsa Race massacre, leading to the destruction of Black Wall Street (Greenwood) and deaths of up to 300 people, was caused by a White female elevator operator who was upset by a Black man. The Oklahoma Historical Society determined that the cause of her distress is that he stepped on her toe. The National Guard assisted in the destruction of the town (Messer et al., 2018).

Many are shocked to learn that the same weaponization of White femininity reaches into the present day, when in 2020, as previously noted, a White woman made a calculated call to 911 to punish a Black man who was birdwatching in New York’s Central Park. On the call she started screaming and said that an African American man was threatening her life, after he merely asked her to leash her dog. Before calling the police, she made sure to threaten him with what they both knew was an invitation to a potentially lethal police confrontation in a powerful example of the way White Women can arm themselves with their identity (Perez-Pena, 2017).

In the graphic classroom anecdote, similarly, the White woman becomes the victim, and by default the Black person becomes the persecutor or the villain (Accapadi, 2007). The distress of a White woman is a cultural trigger that sets off a specific emotional response that can lead to emotional dysregulation and protectionism in some White people (Liebow and Glazer, 2019; Phipps, 2021).

The decision by the White female student to cry in the classroom is a cultural and psychological response made an acceptable strategy to (1) distract from any accusations of personal racism, (2) refocus the center of attention, (3) control the narrative and the room, and (4) seize the position of power, and (5) control the emotions of White men, generally the most powerful people in a setting (Liebow and Glazer, 2019; Butler et al., 2022).

The expectations and treatment of students by instructors has been demonstrated to vary by social class as well as race and gender (Griendling et al., 2023). Further, White female students in particularly may experience marginalization differently based on social class (Silver, 2020; Todd et al., 2023). As such, it can be hypothesized that class may also play a role in these classroom dynamics. However, although research has found that access to extracurricular clubs and groups is impacted by class, it is primarily race and gender that shape the overall student experience, beyond mere access (Silver, 2020). Nonetheless, because White students tend to experience the impact of class differences more keenly than race, they may be more motivated to raise this issue during classroom discussions about race.

White women wielding their status in situations as depicted in the figures are thereby effectively weaponizing their femininity to shield themselves from criticism and control the conversation about race. Figures 1, 2 are designed to help recognize the emotional triggers and emotional dysregulation that are part of the psychological framework that leads to these kinds of outbursts and thereby aid in the understanding and mitigation of such occurrences (DiAngelo, 2011; Armstrong, 2021). Some of the oft hidden or unexamined emotional regulation issues which can arise when confronted by conversations on issues of race include avoidance, narcissism, defensiveness, feeling threatened, and fear (Sue et al., 2010; DiAngelo, 2011).

### 3.1. Creating safe and equitable classroom discussions

There are specific approaches that can be employed to create a safe and equitable classroom space dedicated to learning. The unfolding of events can itself be a teaching tool. Strategies that lecturers and students can use to transform such situations include the following (Sue et al., 2009b, 2010; Williams, 2019; Hochman and Suyemoto, 2020; Williams et al., 2021).

Students:

- *Engage:* White students should allow the discussion about race and racial injustice to unfold. Resist any impulse to become defensive and exit the conversation. Do not derail conversation by refocusing on your own or someone else’s victimhood. Avoid using your authority to stop discussions about race to maintain “peace.”
- *Listen*: No one should sidetrack the conversation to one about social class or gender as this will devalue the experiences of racialized people who are living daily with systemic racism.
- *Center* the voices of students of color. If you are White and find yourself in a conversation about race, WAIT (Why Am I Talking?) Ask yourself if you should be planted in the center of the conversation. Ask yourself if the topic is about you or should be about you at all (e.g., Kuo et al., 2022).
- *Accept criticism*: Discussing the facts about racial injustice should not be seen as a personal attack on your character, your family, or yourself personally. Discussions about race are a prerequisite for greater empathy, self-growth, and true education about systemic injustice.
- *Empathize*: Understand that your personal experiences may be different from someone of a different race. Cultivate compassion toward those who are marginalized by society and try to understand their stories.

Professors can additionally:

- *Regulate* White women attempting to center themselves with displays of emotional distress. Invite these students to step outside the class until they feel ready to re-engage. This disempowers them by removing them from center stage and shows your faith in their ability to regain control of their emotions without assistance.
- *Correct* any students who advance false stereotypes, make racist statements, or work to derail conversations about racial justice; they should be gently corrected during the class as a learning exercise for everyone.
- *Check-in* with any affected students individually after class or during office hours to show that you care, make them feel heard, and gain valuable feedback that you can use to improve the classroom experience.

For instructors, it is important to understand that when issues related to race are discussed in class, expect disruptions to occur and be prepared to defuse the situation from the start by describing in advance the types of dynamics (e.g., outlined in this piece) that can occur. Some White students may never have been involved in a discussion about race or have even thought through the implications of being socialized as a White person (Todd et al., 2023). If students shift the focus from the material and use the class as a forum to deny that Whiteness confers privilege, or even imply this, it is a disservice to students because ultimately the ugly implication would be that people of color are disadvantaged because they are inferior. The instructor should therefore not allow the students to control the class through displays of emotion (Sue et al., 2009b). These conversations can be difficult, and so it may help for instructors to work to increase their own capacity for interracial dialogue (see Williams et al., 2022 for practical exercises to facilitate personal growth).

Professors can also preemptively prevent many such issues by adding material to their syllabi about expectations for classroom behaviors, such as the importance of prioritizing (centering) the voices of people of color when issues of racism are discussed and mutually respectful behavior. Adopting these suggestions should help in facilitating equitable discussions about racial injustice. This will not be easy, as some students will be uncomfortable and may even complain, but this can all be used as an educational opportunity, ultimately fostering greater racial aid in creating harmony and promoting allyship (Liebow and Glazer, 2019; Williams et al., 2020).

## 4. Conclusion

It is crucial to continue researching the complex ways in which race and gender intersect and impact the lives of individuals (Armstrong, 2021; Butler et al., 2022). This power and privilege become evident in the societal deference granted to specific emotional states and experiences that are unique to White women (Armstrong, 2021; Phipps, 2021). Although there are many media reports of this occurring, there is very little contemporary scholarship on the extent, psychology, situational awareness, and deliberation White women go through when exercising these privileged behaviors (Accapadi, 2007; Blow, 2020; Armstrong, 2021).

The issues described are not universal truths, but rather cultural phenomena focused on American and Canadian society. Further research is needed to address the ways in which race and femininity intersect, and the impact that this intersection has on the lived experiences of both women of color and White women broadly (Armstrong, 2021). More research is also needed on the intersection of other identities as well (e.g., religion, age, income, disability, nationality). The escalation of conflict when a White woman uses emotional vulnerability to inflame the passions of onlookers can have life-threatening consequences (Armstrong, 2021; Butler et al., 2022). It is therefore particularly important to dismantle the racism and sexism embedded in the myth of White feminine victimhood to create a more equitable and just society.

## Data availability statement

The original contributions presented in the study are included in the article/supplementary material, further inquiries can be directed to the corresponding author.

## Author contributions

All authors listed have made a substantial, direct, and intellectual contribution to the work and approved it for publication.

## Funding

This research was undertaken, in part, thanks to funding from the Canada Research Chairs Program, Canadian Institutes of Health Research (CIHR) grant number 950-232127 (PI MW).

## Conflict of interest

The authors declare that the research was conducted in the absence of any commercial or financial relationships that could be construed as a potential conflict of interest.

## Publisher’s note

All claims expressed in this article are solely those of the authors and do not necessarily represent those of their affiliated organizations, or those of the publisher, the editors and the reviewers. Any product that may be evaluated in this article, or claim that may be made by its manufacturer, is not guaranteed or endorsed by the publisher.
